# Carbon in Commercially Pure Titanium

**DOI:** 10.3390/ma16020711

**Published:** 2023-01-11

**Authors:** Agnieszka Szkliniarz, Wojciech Szkliniarz

**Affiliations:** 1 Faculty of Materials Engineering, Silesian University of Technology, 44-100 Gliwice, Poland; 2 University Zone of Material Innovations, Silesian University of Technology, 44-100 Gliwice, Poland

**Keywords:** titanium with carbon, titanium alloys, crystallization, microstructure, mechanical properties

## Abstract

In a way so far unmatched in any single study, this paper presents the complex characteristics of commercially pure titanium (CP-Ti) containing 0.2 wt.% carbon, which is significantly above the carbon level in commonly used titanium alloys, while at the same time being the maximum permitted content in light of the recommendations in force. It has been demonstrated that the addition of carbon in CP-Ti can have many positive impacts. The investigated Ti-0.2C alloy was produced in a cold-copper crucible induction vacuum furnace and processed into a 12 mm diameter bar by hot rolling. The structure and properties of the Ti-0.2C alloy were compared to those of an CP-Ti Grade 1 produced and processed under the same technical conditions. The addition of 0.2 wt.% carbon to CP-Ti has been found to change the course of the crystallization process, the course and temperatures of phase transformations, and the values of lattice parameters; reduce susceptibility to grain growth; and create the possibility for additional hardening during solution treatment and aging. At the same time, it results in an assumed improvement in properties by increasing the tensile strength and yield strength, hardness, creep and oxidation resistance, and abrasive wear. It has a negative effect but is still within the acceptable range on impact strength and susceptibility to hot and cold deformation.

## 1. Introduction

Carbon is present in commercially pure titanium (CP-Ti) and in all commercially available titanium alloys as a technological admixture by passing from the charge during the melting. At present, it is no longer considered as contamination of titanium and titanium alloys and is increasingly used as an additive with a very different and increasingly broad spectrum of impacts. The presence and form of carbon in titanium and titanium alloys is determined by its solubility in different titanium phases. The solubility of carbon in β-Ti is merely 0.14 wt.% at the eutectic transformation temperature of 1645 °C. With the decrease in temperature, the solubility slightly decreases to approximately 0.1 wt.% at 920 °C. The solubility of carbon in α-Ti is slightly higher, reaching 0.5 wt.% at 920 °C. At 600 °C, it decreases to 0.2 wt.%, while at room temperature it is approximately 0.08 wt.% [1,2]. In alloys with carbon content above 0.1 wt.%, the portion of carbon not dissolved in α-Ti and β-Ti occurs in the form of titanium carbide TiC.

The first information on the effect of carbon on the properties of CP-Ti and binary alloys dates back to the 1950s [3,4,5]. It was then found that carbon added to the then-produced titanium and titanium alloys in the amount of up to 0.5 wt.% and improved their strength and hardness, while significantly reducing their plastic properties. As a result, carbon and other interstitial elements (oxygen, nitrogen, and hydrogen) became to be considered as contamination, the content of which should be controlled so that it did not exceed 0.2 wt.%. It is only since 2000 that there has been a renewed interest in using carbon as a component to be added to improve the strength of titanium and titanium alloys, while also paying greater attention to other aspects of its positive impact [6,7,8,9,10]. Initially, it was mainly focused on biomedical alloys in the pseudo-β and β groups where detrimental vanadium was replaced by iron and chromium, and the deficiency in the strength of these alloys was compensated by adding oxygen and carbon. A small addition of carbon in these alloys resulted in an increase in the efficiency of aging [11], an increase in structural stability [12], an increase in creep resistance [13], grain refinement, a significant increase in plasticity [14] and sometimes a reduction in plasticity [15,16], and a reduction in fatigue strength [17] or in another case its improvement [18].

Since the global economic crisis, which has recently been exacerbated by the pandemic and the war in Ukraine, looking for cheaper, more available, and lighter substitutes for alloying elements to be used in titanium alloys has begun. In addition to chromium, iron, and manganese, the group of substitutes that meets these criteria also includes interstitial elements, in particular, carbon, which may be a desirable addition also in other groups of titanium alloys [19,20,21,22], including in the newest and most forward-looking group of TiAl intermetallic alloys, where the addition of carbon results in the expected improvement in creep resistance [23,24,25].

The effect of carbon varies between titanium alloy groups, due to, among other things, the fact that alloying components of different natures that are present in titanium alloys (i.e., stabilizing the α phase, stabilizing the β phase, or neutral) have very high impact on the solubility and the form of carbon in titanium alloys, determining which part of carbon is present in the α-Ti- and/or β-Ti-based interstitial solid solutions and which one is present in the form of non-stoichiometric titanium carbide TiC_x_. Given that even a low content of carbon may result in an unacceptable reduction in the plasticity of titanium and titanium alloys, the carbon content in commercial titanium alloys is limited to within 0.02 ÷ 0.10 wt.%, typically 0.05 ÷ 0.08 wt.% [26,27,28].

This paper presents comprehensively the effect of the maximum allowed carbon content based on the current recommendations (0.2 wt.%) on the microstructure and properties of CP-Ti Grade 1 produced in a cold-copper crucible induction vacuum furnace, deformed by hot rolling, and heat-treated under standard conditions. In the absence of other alloying components, the possible improvement or reduction in properties of CP-Ti with carbon addition may only be the result of the presence of carbon. This work determines, among other things, the effect of 0.2 wt.% carbon on the course of the crystallization process, the course and temperature of phase transformations; the values of lattice parameters; the structure, strength, and plastic properties, hardness, impact strength, oxidation and creep resistance; and abrasive wear of CP-Ti, as well as its susceptibility to heat treatment hardening and plastic formability at room and elevated temperatures of CP-Ti. The positive, negative, and neutral effects of the carbon content in CP-Ti were identified.

## 2. Materials and Methods

**Figure 1:**
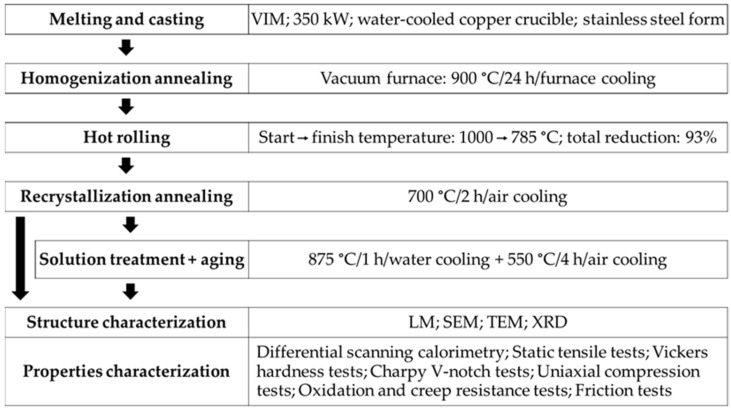
Manufacturing and testing methods of researched alloys.

Alloys manufacturing and testing methods are shown schematically in Figure 1. The researched alloys were melted in a vacuum induction furnace with a cold copper crucible. Titanium with 0.2 wt.% of carbon in comparison with pure titanium (Grade 1, 3.7025, Hempel Special Metals sp zo.o., Sosnowiec, Poland) was studied. Carbon was introduced in the form of anthracite (94% C, as raw materials ~50 mm pieces). Re-melted materials were cast via gravity casting to ingots 40 mm in diameter and 350 mm in length and were homogenized in the vacuum furnace at 900 °C/24 h and then hot rolled. The samples were rolled to a total strain of 93% reduction. Interstitial elements of the experimental alloys are shown in Table 1. Chemical analysis of O and C was carried out using ONH836 and Leco CS844 analyzers. The obtained bars of 12 mm in diameter were subjected to final heat treatment conducted under standard conditions for reference CP titanium (without additional carbon content), which consisted of annealing (700 °C/2 h/air). Since CP-Ti as well as single-phase alloys are used only in the annealed state, the parameters of the strengthening heat treatment have been specially selected. The parameters of solution heat treatment [29] and aging were, respectively, 875 °C/1 h/water and 550 °C/4 h/air.

Standard metallographic techniques were employed for microstructural observation using optical and scanning electron microscopes and finally, etching was carried out with Kroll’s reagent. For transmission electron microscopy, specimens were produced by cutting 3 mm discs from each sample using electrical discharge machining, manually polishing to 200 µm thickness, and electrolytic polishing to transparency using 60 vol.% methanol, 35 vol.% butanol, and 5 vol.% perchloric acid solution at −30 °C in a twin-jet polisher (Struers Tenupol, Ballerup, Denmark). During polishing, the voltage was maintained at 20 V. For quantitative analysis of microstructure elements, Met-Ilo software 14.0 was used.

The analysis of the phase composition was carried out using a JEOL JDX-7S X-ray diffractometer. Lattice parameters can be precisely measured using X-ray diffraction methods. Transformation temperature was determined by differential scanning calorimetry (DSC) methods. Hardness measurements corresponding to Vickers HV1 were performed using a universal hardness testing machine, Zwick. Mechanical properties were tested, at room and elevated temperature, on bar specimens using a Zwick/Roell Z100 machine according to PN-EN ISO 6892-1:2010. The uniaxial compression tests at a rate of 1.0 s^−1^ up to the strain of 1.0 at room and elevated temperatures were performed with Gleeble HDS-V40 and Gleeble 3800 simulators. The short-term creep tests were carried out according to ASTM 139. The oxidation resistance tests were conducted by the conventional isobaric–isothermal method using a GDTD16 thermo-gravimetric analyzer. Kinetic measurements were taken at 650 °C in the atmosphere of technical oxygen passing at a rate of 1 l/h. Experimental friction tests were carried out on pin-on-disc testers in sliding motion with a sliding velocity of 0.5 m/s, CP-Ti as a counter-sample, and commercially dry conditions.

## 3. Results and Discussion

### 3.1. Alloys Phase Composition

**Figure 2:**
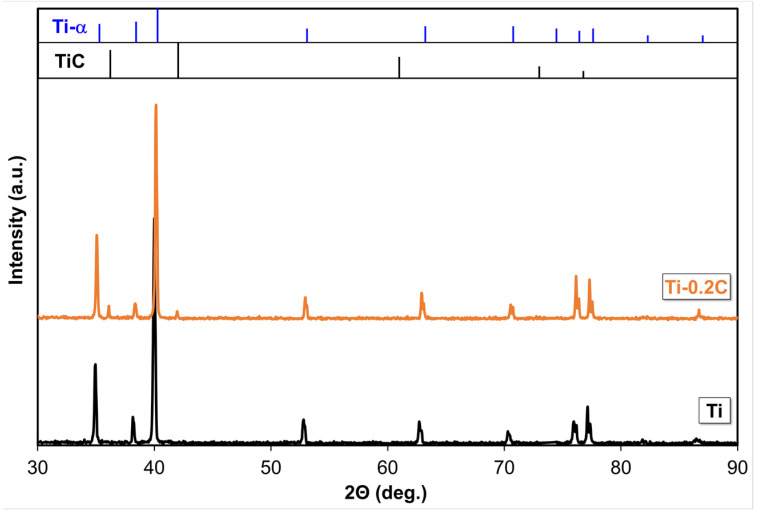
X-ray diffraction patterns (XRD) of CP-Ti and the Ti-0.2C alloy.

**Figure 3:**
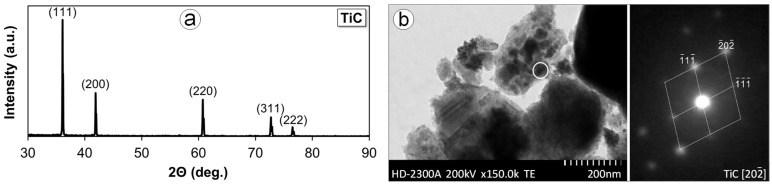
Structure of isolated carbides occurring in the Ti-0.2C alloy with X-ray diffraction pattern (XRD) (**a**) and electron diffraction pattern from selected area (SAED) (**b**).

The analysis of the phase composition test results for the produced CP-Ti and Ti-0.2C, presented in the form of a complex diffraction pattern in Figure 2, has revealed the presence of mainly the α phase in the structure of these alloys. Only the diffraction pattern for the Ti-0.2C alloy shows single weak reflections from TiC carbide that prove the presence of this phase in the structure of the alloys. However, the presence of TiC carbides in the Ti-0.2C alloy was clearly confirmed by tests on powdered isolate carried out independently by X-ray diffraction (Figure 3a) and electron diffraction (Figure 3b).

Carbon, as an alpha stabilizer, prefers to dissolve in alpha titanium but on the contrary, in comparison with other interstitial elements, such as oxygen or nitrogen, has very limited solubility in alpha titanium and forms a peritectoid system. The presence of carbon in titanium alloys increases the values of lattice constants in the α phase (Table 2). As this increase concerns c_α_ rather than a_α_, it results in an increase in the c_α_/a_α_ ratio (Table 2) from 1.5864 in CP-Ti to 1.5874 in the Ti-0.2C alloy. According to Ogden and Jaffee [3], carbon, compared to oxygen and nitrogen, has the greatest effect on the increase in the a and c lattice parameters of the hexagonal close-packed alpha phase, and thus the c/a ratio. The maximum permitted limit of the c/a ratio, suggested for maintaining good hot formability, equals 1.595, and the value of the c/a ratio, obtained for Ti-0.2C, complies with this condition.

Carbon, classified as an alpha stabilizer, increases the transformation temperature of titanium. In CP-Ti, the α→β transformation finish temperature is 890 °C, whereas in Ti-0.2C, this temperature rises to 920 °C (Table 2). Carbon has a much greater effect on raising the transformation temperature of titanium alloys. Zhang et al. [30] showed that the addition of 0.23 wt.% carbon to Ti-5.6Al-4.8Sn-2Zr-1Mo-0.35Si-0.7Nd increased the α + β→β transus temperature by 155 °C. Solonina and Ulyakova [5] reported that the addition of 0.25% C to the Ti-6Al-3Zr-2Mo alloy raised the transformation temperature by 100 °C. In this case, the temperature of the polymorphous transformation was determined by quenching the samples and examining the microstructure in a light microscope. 

### 3.2. Alloys Microstructure

The structure of the tested CP-Ti and the Ti-0.2C alloy and the morphology of the carbide phase are the total impacts of the processes and transformations that occur under crystallization conditions, the plastic working, as well as the intermediate and final heat treatment and, respectively, the presence of carbon.

**Figure 4:**
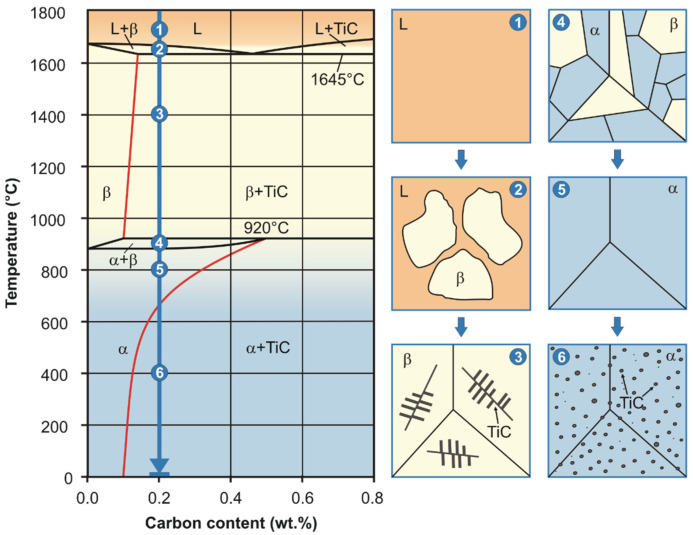
Scheme of microstructure changes during crystallization process of the Ti-0.2C alloy presented against the background of the Ti-C phase diagram.

**Figure 5:**
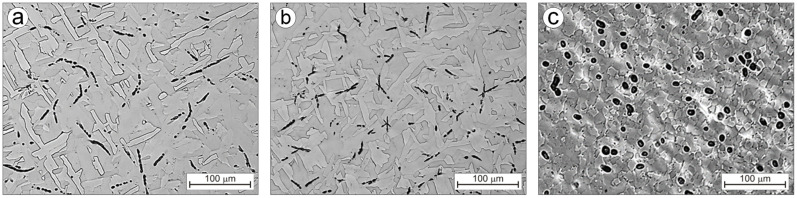
Microstructure of the Ti-0.2C alloy after casting (**a**), homogenization (**b**), and hot rolling (**c**).

The diagram presented in Figure 4 shows that the formation of the Ti-0.2C alloy microstructure begins with the crystallization process starting at approximately 1660 °C. Finally, the following should be present in the microstructure of the cast Ti-0.2C alloy at room temperature following the sequence of the L→L + β→β + TiC→α + β→α→α + TiC transformations (point 1–6 in Figure 4) resulting from the Ti-C equilibrium system: the α phase, which is an interstitial solid solution of carbon in the α-Ti, and the dispersive TiC carbide precipitates with a relative volume estimated at approximately 0.75% based on the lever rule, precipitated from the α phase at below 660 °C due to a change in solubility of carbon in this phase with temperature. However, in the real microstructure of the cast Ti-0.2C alloy (Figure 5a), instead of dispersive TiC carbides, large carbides of irregular shape and more than twice the occupied area fraction of 1.51% (Table 3) are present. These facts clearly indicate that the conditions for the crystallization of the Ti-0.2C alloy in the metal ingot mold are far from equilibrium conditions.

Present in the microstructure of the Ti-0.2C alloy after casting, TiC carbides have the shape of short rods of 1 ÷ 3 μm in diameter with a length of a few to several μm (Figure 5a). They form characteristic chains and are rather evenly distributed at random locations within the matrix. The mutual distribution of the phase crystals, as a matrix of alloys, and carbides (Figure 5a) indicates that carbides are not natural obstacles to the growth of the α phase crystals that occurred as a result of the β→α transformation.

The morphology of carbides present in the Ti-0.2C alloy changes during the homogenization annealing, plastic working, recrystallization annealing, and final hardening heat treatment processes.

The use of long-term homogenization annealing of the Ti-0.2C alloy after the crystallization results in noticeable, although small changes in the morphology of carbides in its microstructure (Figure 5b). The characteristics of the carbide phase in Table 3 show that following the homogenization annealing the average fraction of the area occupied by carbides A_A_ and the average area of the plane section of carbides A decrease. This means that carbides are partially dissolved during the homogenization annealing, and the carbon contained in them, is transferred to the α solid solution, which is the matrix.

**Figure 6:**
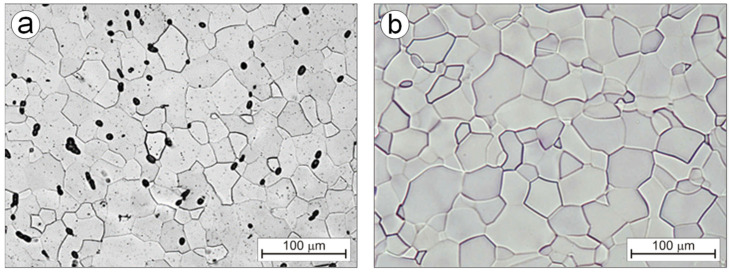
Microstructure of the Ti-0.2C alloy (**a**) and CP-Ti (**b**) after recrystallization annealing.

Carbides that form characteristic chains are broken into pieces of shapes similar to the spheroidal one during the multi-pass hot rolling of the Ti-0.2C alloy, as evidenced by a change in the carbide shape index from approximately 0.5 in the as-homogenized condition to above 0.9 in the as-hot rolled condition (Table 3). They have larger sizes and a higher occupied area fraction compared to the carbides present in the microstructure before rolling (Table 3). Occurring mainly at the boundaries of partially recrystallized areas with undeveloped grain boundaries (Figure 5c), carbides hinder the structure recovery processes for the deformed Ti-0.2C alloy. Therefore, additional recrystallization annealing was carried out to complete the recrystallization process and ensure the full development of the grain structure of the Ti-0.2C alloy. The microstructure of the annealed Ti-0.2C alloy consists of equiaxed α phase grains, large globular carbides at the grain boundaries, and small carbides within the grains (Figure 6a). The annealing is accompanied by a slight increase in the α phase grain growth compared to the grain in the as-hot rolled condition (Figure 5c and Figure 6b). From the comparison of the microstructures of the Ti-0.2C alloy and CP-Ti, presented in Figure 6a,b, it appears that carbides occurring at the grain boundaries during the recrystallization annealing are an effective barrier to the grain growth.

One of the important features of titanium alloys, which distinguishes them from other construction materials, is the hardening capability during heat treatment processes, resulting in an increase in strength properties by even up to 50% [26,27,28]. Unfortunately, the hardening heat treatment, which consists of combined supercooling and aging treatments, is effective only to those titanium alloys for which supercooling of the high-temperature β phase to room temperature is possible. This applies only to α + β and pseudo-β alloys and, to a limited extent, to pseudo-α alloys. For this reason, CP-Ti as well as α and β single-phase titanium alloys are used only in the as-annealed condition. The presence of carbon—the α phase stabilizing element—in these alloys also does not create the possibility of supercooling the β phase; however, due to the temperature related solubility of this element in the α phase (Figure 4), it allows another hardening heat treatment consisting of combined solution and aging treatments to be used.

**Figure 7:**
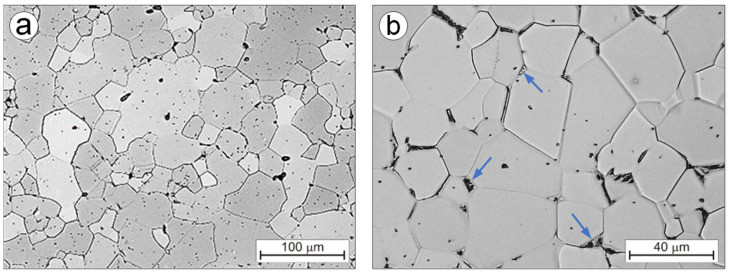
Microstructure of the Ti-0.2C alloy after solution treatment at different magnification (**a**,**b**).

**Figure 8:**
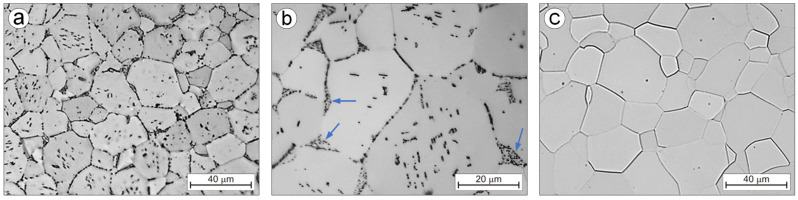
Microstructure of Ti-0.2C alloys (**a**,**b**) and CP-Ti (**c**) after solution treatment and aging.

**Figure 9:**
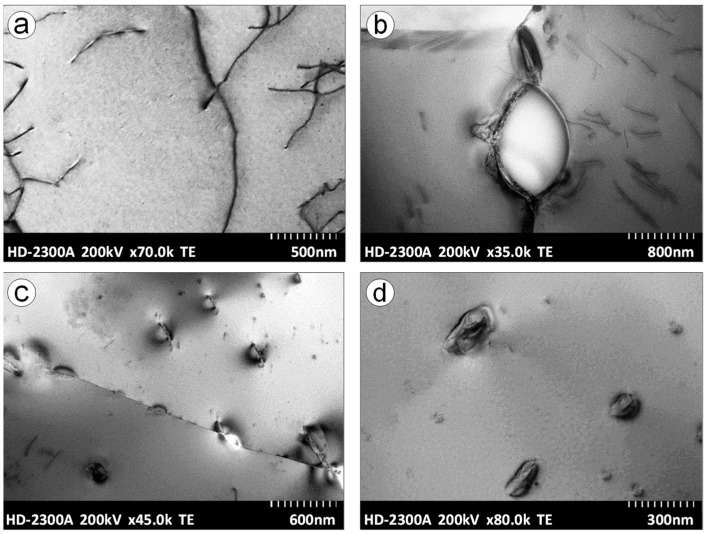
Substructure of CP-Ti (**a**) and the Ti-0.2C alloy (**b**–**d**) after solution treatment and aging.

When soaking the Ti-0.2C alloy until solution at a temperature above the carbon solubility limit in the α phase (875 °C), the carbide dissolving processes and carbon transfer to the α solution as well as partial α→β phase transformation take place. The fraction of the area occupied by A_A_ carbides following a solution decreases from 1.16% to 0.42% (Table 3). The microstructure of the alloy solution treated in water consists of the residues of undissolved carbides present at the boundaries and within the equiaxed grains of the carbon-supersaturated α phase (Figure 7a) and the martensitic α’ phase, formed as a result of martensitic β→α’ transformation and present in the form of island clusters at the junction of three grains (Figure 7b). During the aging of the solution-treated alloy, the precipitation of carbides from the carbon-supersaturated α phase and the martensitic α’ phase transformation into the balanced α phase take place. After aging, the microstructure of the Ti-0.2C alloy consists of the dispersive carbides that occur mainly at the boundaries and, to a lesser extent, within the α phase grains (Figure 8a) and decomposition products of the island clusters of the former martensitic α’ phase (Figure 8b). In the CP-Ti solution treated and aged under the same conditions, carbides are not present (Figure 8c). It was also confirmed by examinations of an alloy microstructure with transmission electron microscopy—TEM (Figure 9a). The microstructure examinations of the solution-treated and -aged Ti-0.2C alloy by TEM methods revealed the presence of numerous dispersion precipitates of various sizes (Figure 9b–d). They consist of single and incompletely dissolved carbide precipitates located at the grain boundaries (Figure 9b) and numerous and very fine carbides precipitated from the supersaturated matrix, located at the boundaries and within the grains (Figure 9c,d). Most of them are semi-coherent precipitates. Some of the carbide precipitates meet the nanoparticle size criterion (Figure 9d). A mutual interaction between the dispersive precipitates and the grain boundaries and dislocations was also found (Figure 9b,c), which may confirm the active role of carbides in the hardening of the alloy.

### 3.3. Alloys Properties

#### 3.3.1. Mechanical Properties

**Figure 10:**
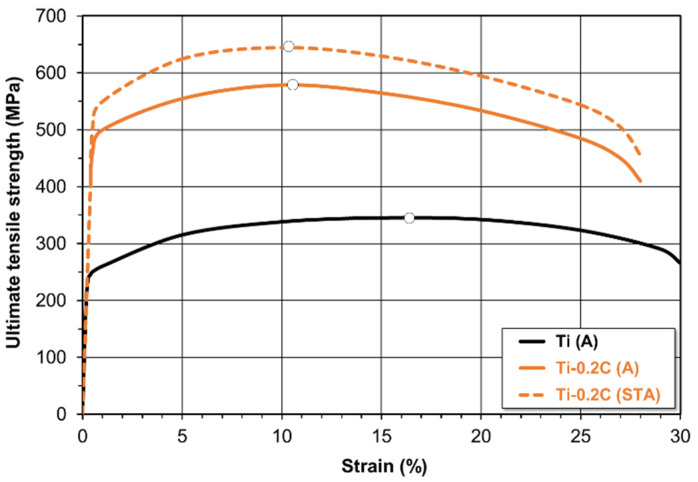
Effect of carbon on the stress–strain characteristics of the CP-Ti.

CP-Ti deforms uniformly to the strain of approximately 16.5%, at which the maximum hardening occurs and the neck forming (reduction in area) process begins. In the Ti-0.2C alloy, the same processes take place after a smaller deformation is applied, slightly above approximately 10.0% (Figure 10). The expected effect of carbon presence in titanium is the increase in strength properties. Table 4 shows the values of ultimate tensile strength (UTS), yield strength (YS), elongation (EL), reduction in area (RA), Young’s modulus (E), Vickers hardness (HV), and impact energy (CVN) of CP-Ti and the Ti-0.2C alloy after the recrystallization annealing and the solution treatment and aging.

The introduction of 0.2 wt.% carbon to CP-Ti has resulted in an increase in the tensile strength of the Ti-0.2C alloy in the as-annealed condition from 345 to 579 MPa and the yield strength from 245 to 496 MPa, while maintaining almost unchanged plastic properties (Table 4). The strength properties of the Ti-0.2C alloy are higher than those of the most durable grade of CP-Ti, i.e., Grade 4 (Table 4). The addition of 0.2% C to iodide titanium [3] results in a lower strength and higher ductility compared to the tested alloy due to its higher purity. The authors stated that carbon was effective as a strengthener only up to its limit of solubility in the alpha phase. Above this limit, carbon, present as TiC carbides, had no significant effect on the strengthening. Additionally, Ozerov et al. [31] reported that the YS of alloys with carbon (Ti-0.1C and Ti-0.2C) was ~55% higher in comparison with pure titanium. They also showed that cold rolling led to an increase in the YS to 630 and 605 MPa, respectively, with carbon content.

After the application of solution treatment and aging, the tensile strength and yield strength of the Ti-0.2C alloy were increased by another tens of MPa, up to 643 and 554 MPa, respectively (Table 4). The hardening of the Ti-0.2C alloy is accompanied by a slight reduction in plastic properties, e.g., elongation from 29.8% to 28.6% after annealing and to 28.3% after solution treatment and aging. The elongation of the Ti-0.2C alloy, even in the as-hardened condition, is much higher than the elongation of Grade 4 titanium, which has significantly lower strength properties compared to those of the Ti-0.2C alloy (Table 4). There is a consensus of views [7,11,20,30,32,33] that carbon increases the age hardenability of titanium alloys. Yu et al. [34] reported that a slight increase in the content of carbon (from 0.008 to 0.034 wt.%) in the quenched Ti-10V-2Fe-3Al alloy and a decrease in the rate of heating to the aging temperature improves the dispersity of secondary phases formed after quenching and promotes growth in the alloy hardness.

The presence of carbon also has a positive, although slight, effect on the modulus of longitudinal elasticity (Young’s modulus), which is approximately 104–105 GPa in the Ti-0.2C alloy (Table 4).

The hardness of the Ti-0.2C alloy in the as-annealed and as-hardened conditions is much higher than the hardness of carbon-free CP-Ti (Table 4). After the addition of 0.2 wt.% carbon, the hardness of CP-Ti increases by approximately 50 HV in the as-annealed condition and approximately 90 HV in the as-hardened condition.

The information on the adverse effect of carbon on the impact strength of titanium alloys [3,26,27,35] is confirmed by the results of investigations in Table 4. It follows from them that the presence of carbon has a negative effect on the impact energy CVN of the annealed and hardened alloy, as determined by the impact bending tests. The impact energy of the Ti-0.2C alloy, although clearly lower than that of carbon-free CP-Ti, meets the minimum requirements specified for CP-Ti in the standards [36].

**Figure 11:**
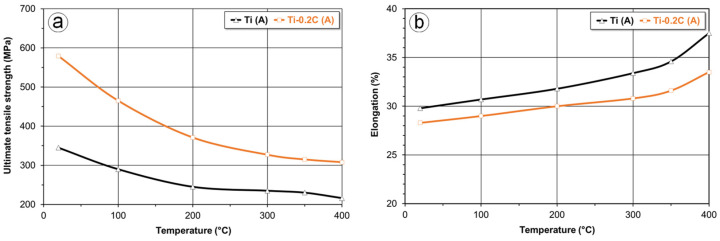
Effect of carbon and temperature on the ultimate tensile strength (**a**) and elongation (**b**) of CP-Ti.

Compared to carbon-free CP-Ti, the Ti-0.2C titanium alloy with carbon has a higher tensile strength also at an elevated temperature (Figure 11a). Although the tensile strength of the Ti-0.2C alloy decreases with temperature rise more than the tensile strength of CP-Ti, it is still higher than that of CP-Ti by more than 100 MPa at 400 °C, i.e., the temperature by 100 °C higher than the maximum application temperature of CP-Ti. The reduction in tensile strength of the Ti-0.2C alloy with the temperature rise is accompanied by an increase in the elongation, which is higher, the higher temperature at which the test is carried out (Figure 11b).

#### 3.3.2. Plastic Formability

The higher strength properties of the Ti-0.2C alloy compared to those of CP-Ti at room (Table 4) and elevated (Figure 11a) temperatures indicate that higher resistance to forming should be expected during the plastic deformation of titanium alloys with carbon. The comparative studies of susceptibility to forming for the Ti-0.2C alloy and CP-Ti, conducted under uniaxial compression conditions, fully confirmed these predictions.

**Figure 12:**
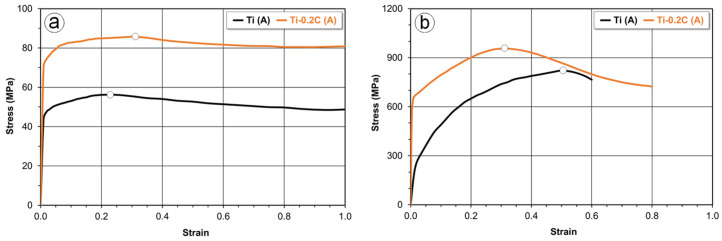
Effect of carbon on the flow curves of CP-Ti at 890 °C (**a**) and at room temperature (**b**).

A characteristic increase in the yield stress until the maximum level followed by a monotonic decrease is observed on the flow curves for CP-Ti and the Ti-0.2C alloy deformed at 890 °C, which is slightly lower than the α→β transformation finish temperature of 920 °C, proving the dynamic recrystallization in the deformed alloys (Figure 12a). In general, the presence of carbon increases the maximum yield stress and the strain at which it occurs (Figure 12a). In CP-Ti deformed at 890 °C, the yield stress reaches the maximum value of 57 MPa at a strain of approximately 0.23. In the Ti-0.2C alloy, the maximum yield stress of 86 MPa, which is 50% higher, was obtained at a strain of approximately 0.30.

It follows from the flow curves for CP-Ti and the Ti-0.2C alloy (Figure 12b) recorded during the compression test conducted at room temperature at a strain rate of 1.0 s^−1^ that CP-Ti can be cold deformed to a strain of 0.5 without cracking. At this strain, CP-Ti is hardened to the maximum stress of 882 MPa. After this strain is exceeded, cracks appear on the CP-Ti surface, and the progressive cohesion loss is reflected as a decrease in stress in the flow curve (Figure 12b). In the cold-deformed Ti-0.2C alloy, the cohesion loss process initiates at higher stresses and lower strains of 956 MPa and 0.31, respectively.

#### 3.3.3. Creep Resistance

Among currently produced titanium alloys for service under creep conditions, the most commonly used are pseudo-α, α, and α + β alloys. CP-Ti and pseudo-β and β alloys are used very rarely. The best of them—pseudo-α alloys—can be used at a temperature not exceeding 600 °C, and an important component of these alloys is silicon added in quantities of up to 0.5% [37,38,39]. The improvement in creep resistance of silicon-containing titanium alloys is associated with the solid solution strengthening (the Cottrell atmospheres formed around the silicon atoms hinder the movement of dislocation) and the precipitation hardening (at the silicon content exceeding the solubility limit in the α phase, fine-dispersion Ti_5_Si_3_ and Ti_2_Si intermetallic phases inhibiting the dislocation slip are formed during aging) [37,39]. Since these are the same mechanisms that determine the hardening of CP-Ti containing carbon [3,40,41,42], there are indications that carbon can be used as a component to increase the suitability of CP-Ti for service under elevated temperature and stress conditions.

**Figure 13:**
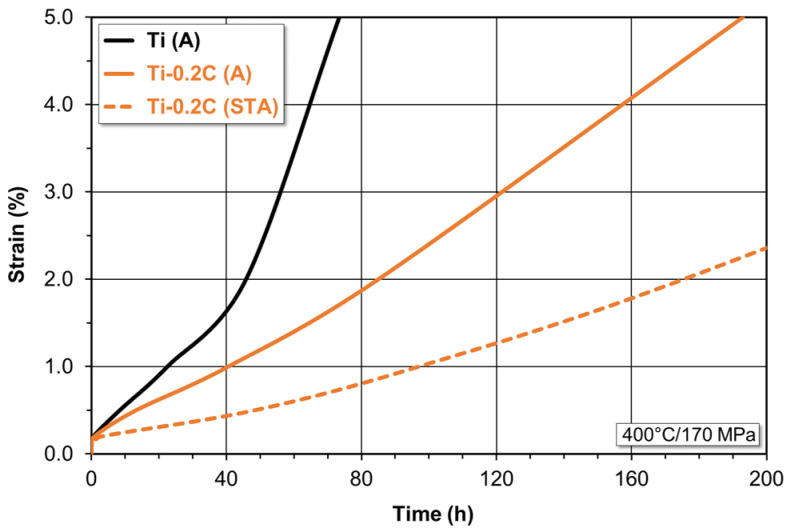
Effect of carbon on the creep curves of CP-Ti.

The comparative short-term creep tests (up to approximately 200 h) of CP-Ti and the Ti-0.2C alloy in the as-annealed and as-hardened condition produced as a result of combined solution treatment and aging have shown that after a very short period of hardening, characteristic of the primary creep, the alloys enter the secondary creep stage characterized by a constant creep rate, and then, after several dozen hours, the tertiary creep stage (Figure 13).

After 0.6 h of testing and reaching the strain of 0.2%, the annealed CP-Ti subjected to the creep test at 400 °C and 170 MPa enters the secondary creep stage at the rate of 3.55 × 10^−4^s^−1^ (Figure 13). At this creep rate, CP-Ti reaches the strain of 0.5 and 1.0% after 8.4 and 22.5 h, respectively. After 38 h of testing and reaching the strain of 1.6%, it enters the rapid transient creep stage. The introduction of 0.2 wt.% carbon to CP-Ti has changed the course of the creep process. The annealed Ti-0.2C alloy enters the steady–state creep at almost twice the lower rate equal to 1.71 × 10^−4^s^−1^ after 59 h of testing and at the strain of 0.2%. At this creep rate, the Ti-0.2C alloy in the as-annealed condition reaches the strain of 0.5 and 1.0% after 13.2 and 40.5 h, respectively. Entering the transient creep stage takes place after 60 h of testing and at the strain of 1.4% (Figure 13). The hardening heat treatment of the Ti-0.2C alloy results in further positive changes in the creep process for this alloy (Figure 13). The hardened Ti-0.2C alloy enters the steady-state creep at almost 2.5 lower rate compared to that of the annealed alloy equal to 0.67 × 10^−4^s^−1^ after 2.9 h of testing and at the strain of 0.2%. At this creep rate, the Ti-0.2C alloy in the as-hardened condition reaches the strain of 0.5% after 48.3 h. Entering the very low transient creep stage takes place after 65 h of testing and at the strain of 0.6% (Figure 13).

The analysis of the creep curves for CP-Ti and the Ti-0.2C alloy in Figure 13 shows that the addition of carbon improves the creep resistance in the as-annealed condition, and even more in the as-hardened condition. This takes place, among other things, by the reduction in the rate and extension of the time of the secondary creep stage, the delay in transition to the primary creep stage, or extension of the time corresponding to the determined values of the strain that accompanies creep. The improvement in creep resistance of the Ti-0.2C alloy should be associated with the solid solution and precipitation hardening mechanisms. They result from the presence of carbon in the α interstitial solution and in titanium carbides.

#### 3.3.4. Oxidation Resistance

The surface of CP-Ti and titanium alloys exposed to the oxidizing environment is covered by a very thin and passive layer of TiO_2_ titanium oxide, which provides protection against further oxidation of the alloy surface and exceptional resistance in various corrosive environments. However, at elevated temperatures, the TiO_2_ layer loses its protective properties due to reduced adhesion to the substrate and increased porosity.

The analysis of the literature data shows that the effective ways to improve the oxidation resistance of titanium and titanium alloys include: the introduction of alloying components in the form of aluminum, niobium, molybdenum, tungsten, and silicon [35]; the modification of the chemical composition of the surface layer by surface treatments; the application of special protective coatings [26,27,28,43]; and the reduction in oxygen diffusion activity [28,44,45], e.g., by limiting its solubility in different phases that occur in titanium alloys. The solubility of oxygen in α-Ti is approximately 33 at.% and does not change much with temperature, while in β-Ti, it is much lower and changes with temperature between 4 and 8 at.%. [27,28]. It appears that the presence of carbon in titanium and titanium alloys can affect the reduction in oxygen diffusion activity. The interstitial oxygen and carbon atoms occupy the same octahedral holes in α-Ti and tetrahedral holes in β-Ti. Thus, appearing as interstitial solid solutions, carbon will reduce the solubility and oxygen diffusion activity. In turn, when occurring in TiC carbides, it will also reduce the oxygen diffusion activity by intercepting the oxygen atoms from the matrix and locating them in unsettled nodes in the carbon sublattice of the carbides [17,28]. There are, therefore, real indications that carbon has a positive role in improving the oxidation resistance of CP-Ti, the only matrix of which is the α phase with high carbon solubility.

**Figure 14:**
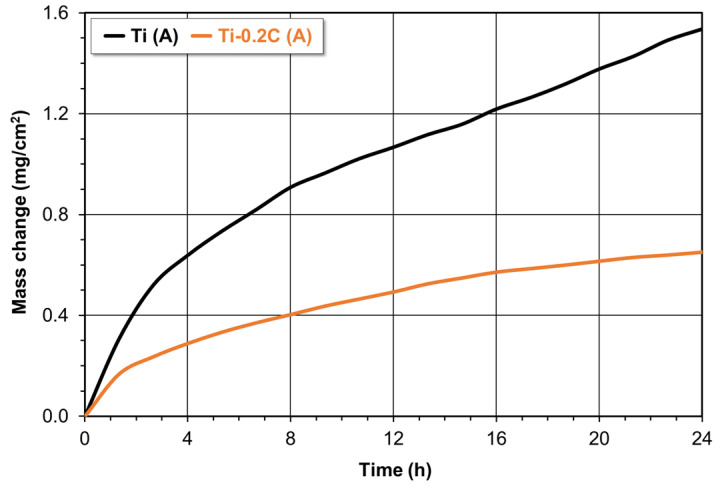
Effect of carbon on the oxidation kinetics of CP-Ti.

The results of the studies on the isothermal oxidation kinetics of annealed CP-Ti and the Ti-0.2C alloy are presented in Figure 14. The oxidation curves representing the mass gain per unit area of a sample as a function of the oxidation time (up to 24 h) show that the initial period of rapid mass growth is followed by a period of stable oxidation with low mass gains (Figure 14). The oxidation curves for CP-Ti and the Ti-0.2C alloy are consistent with the parabolic oxidation law as evidenced by the high values of correlation coefficients R^2^—0.9928 and 0.9909, respectively, indicating the compliance of the obtained experimental results with those described by the Pilling–Bedworth equation. The introduction of 0.2 wt.% carbon into CP-Ti with poor oxidation resistance causes its significant improvement, as evidenced by the oxidation rate constant decreasing from 2.64 × 10^−11^ to 5.05 × 10^−12^ g^2^·cm^−4^·s^−1^. In regard to the stable oxidation, the mass gain of the Ti-0.2C alloy is more than twice the mass gain associated with the oxidation of CP-Ti, and this variation increases with the oxidation time (Figure 14).

#### 3.3.5. Abrasive Wear Resistance

With relatively high resistance, CP-Ti and titanium alloys have relatively low hardness and abrasion resistance. Hence, titanium and titanium alloys are susceptible to rubbing, sticking to mating surfaces, or even getting locked together. At the same time, they often show a high and unstable value of the coefficient of friction, which limits their application in friction systems characterized by high contact loads and the occurrence of sliding friction between mating surfaces [46,47,48]. The hardness test results in Table 4 indicate that carbon, which has a positive effect on the increase in hardness of CP-Ti, should also effectively improve its resistance to abrasive wear.

The changes in values of the coefficient of friction as a function of the friction pair presented in Table 5 indicate that CP-Ti in combination with CP-Ti is unstable under abrasion conditions. The value of the coefficient of friction in this combination is subject to fluctuations and shows a slightly upward trend until it stabilizes after reaching the friction path of at least 150 m. The Ti-0.2C alloy in combination with CP-Ti shows much more stable behavior under abrasion conditions, and the values of the coefficient of friction reveal exceptional stability in the abrasion process. The average value of the coefficient of friction in the CP-Ti/CP-Ti combination is 0.252 (Table 5). In the Ti-0.2C/CP-Ti combination, the average coefficient of friction is lower and equals 0.18. The indication of the positive effect of carbon on an increase in the abrasive wear resistance of the Ti-0.2C alloy in combination with CP-Ti is also the significant decrease in loss of mass of the abrasive samples from 0.02 to 0.007 g, measured on the friction path of 250 m (Table 5).

## 4. Conclusions

Based on the conducted research on the suitability of using carbon as a component to improve the properties of CP-Ti, the following conclusions can be drawn:

1. Carbon added to commercially pure titanium (CP-Ti) in the amount of 0.2 wt.% is within the maximum solubility limits in the α-Ti-based interstitial solid solution, while in the remaining part of titanium carbides, it increases approximately 0.3% of the values of lattice constants of the phases, and the α→β transformation temperature from 890 to 920 °C reduces the susceptibility to grain growth and creates the possibility for additional hardening of approximately 11.0% in the combined solution treatment and aging processes.
2. The addition of 0.2 wt.% carbon to CP-Ti results in the expected improvement in its properties by an increase in the tensile strength by approximately 85%, the yield strength by approximately 125%, and the hardness by approximately 55%, a slight increase in the Young’s modulus from 103 to 105 MPa, and a significant increase in the creep resistance (a 2.5-times decrease in the steady-state creep rate), oxidation resistance (by about 80% reduction in the value of the oxidation rate constant), and abrasive wear resistance (by an approximately 28.5% reduction in the value of the coefficient of friction. At the same time, it does not cause a significant deterioration of plasticity measured during the static tensile test (by approximately 4–5%). The only negative consequences of the presence of 0.2 wt.% C in CP-Ti is a deterioration of the impact strength and hot as well as cold formability to the acceptable level.
3. The results of the comparison of the phase composition, structure, and most important properties of CP-Ti and the Ti-0.2C alloy with the maximum permitted carbon content of approximately 0.2 wt.%, according to the recommendations in force, presented in the form of positive, negative, and neutral effects of carbon on the various properties of the test alloys and showed that carbon in titanium alloys, at the controlled content of up to approximately 0.2 wt.%, can perform many useful functions.

## Figures and Tables

**Table 1:** Chemical composition of investigated alloys.

Alloy	Elements Content (wt.%)				
	C	O	N	H	Ti
CP-Ti	0.03	0.16	0.03	0.014	Balance
Ti-0.2C	0.26	0.17	0.03	0.015	

**Table 2:** Lattice parameters of phases and beta-transus temperature of investigated alloys.

Parameter	Alloy	
	CP-Ti	Ti-0.2C
a_α_ (nm)	0.2945	0.2952
c_α_ (nm)	0.4672	0.4686
c_α_/a_α_	1.5864	1.5874
a_TiC_ (nm)	-	0.4308
T^α→β^ (°C)	890	920

**Table 3:** Hardness and stereological parameters of carbides in the microstructure of investigated alloys.

Alloy	Condition	Hardness HV	Stereological Parameters of Carbides		
			A_A_ (%)	A (μm^2^)	ξ
Ti-0.2C	Casting	204 ± 6	1.51	10.45	0.62
	Homogenization	230 ± 4	1.20	8.98	0.68
	Hot rolling	191 ± 7	1.36	16.21	0.93
	Recrystallization annealing	205 ± 4	1.16	16.46	0.94
	Solution treatment	212 ± 4	0.42	5.65	0.92
	Solution treatment and aging	244 ± 6	1.08	4.25	0.90

A_A_—area fraction; A—mean area; ξ—shape factor.

**Table 4:** Mechanical properties of investigated alloys.

Alloy	Condition	UTS (MPa)	YS (MPa)	EL (%)	RA (%)	E (GPa)	HV	CVN (J)
CP-Ti	A	345	245	29.8	51.5	103	157	84.5
	STA	355	252	29.2	50.3	103	158	86.0
Ti-0.2C	A	579	496	28.6	48.6	104	205	67.0
	STA	643	554	28.3	46.6	105	244	54.5
Grade 4	A	550	485	15.0	30.0	104	250	20.0

A—annealed; STA—solution treated and aged.

**Table 5:** The coefficient of friction in a friction pair: CP-Ti/CP-Ti and Ti-0.2C/CP-Ti.

Alloy	CP-Ti	Ti-0.2C
Coefficient of friction change	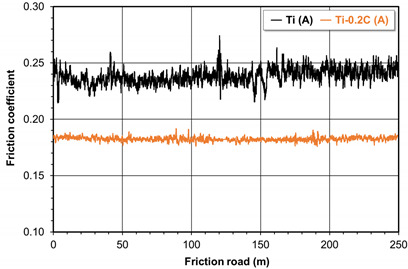	
Coefficient of friction	0.252 ± 0.006	0.180 ± 0.002
Mass loss (g)	0.020	0.007

## Data Availability

Not applicable.
